# The impact of the COVID-19 pandemic on language deserves more attention: evidence from university students in Greece—a research note

**DOI:** 10.1007/s44192-022-00026-x

**Published:** 2022-12-15

**Authors:** Anna Loudovikou, Christos Tsagkaris, Vasiliki Papakosta, Andreas S. Papazoglou, Dimitrios V. Moysidis

**Affiliations:** 1 Department of Applied Linguistics-Neurolinguistics, Aristotle University of Thessaloniki, Thessaloniki, Greece; 2 European Student Think Tank, Public Health and Policy Working Group, 1058 Amsterdam, Netherlands; 3 Faculty of Medicine, University of Crete, Heraklion, Greece; 4 Faculty of Medicine, Aristotle University of Thessaloniki, Thessaloniki, Greece

Dear editor,

A growing amount of scientific evidence shows that the pandemic has affected several domains of mental health among Greek children adolescents. A recent observational study based on questionnaires and conducted by Greek researchers showed a significant increase in anxiety, depression and worsening of quality of life during the pandemic [1]. Another study by Kleinman et al. attempted to investigate whether language comprehension can be changed in a lasting way by external experiences and highlighted that the pandemic has reshaped language processing for the long term, changing how listeners process speech and what they expect from ambiguous input [2]. Furthermore, Marshall et al. explored the potential contribution of natural language processing to analyze how the pandemic may be impacting people’s mental health and well-being [3]. However, these studies examined language from a neurological point of view and perspective, and no study so far has assessed the influence of the pandemic on students’ language/expression, linguistic adoption, and communicative competence. Therefore, the effect of the pandemic and the subsequent disruption of education and socialization on the linguistic abilities of this group is yet to be investigated.

Language as an entity could be compared to living organisms as it functions according to the rules of natural selection. Neuropsychological mechanisms induce the adaptation of spoken languages to emerging challenges, such as financial crises, armed conflicts, social disparities and health crises, in the same manner that sleep, physical performance and appetite are modified to help individuals cope with crisis [4]. The COVID-19 pandemic could not be an exception to how crises affect human linguistic performance. A number of terms and concepts such as pandemic, lockdown, quarantine, click-away, click-inside, rapid-test, post-covid syndrome have infiltrated languages and dialects across the world during the last two years. The same applies to the Greek language, from where the very term "pandemic" stems [5]. Past experience has shown that multidisciplinary research on the linguistic adaptation to crisis can help understand their impact on identity, culture and behavior. Particularly in the context of health crisis, such research can help identify real world needs that can be addressed through education, and health promotion. Young citizens are considered as a high interest group in this field, because they tend to receive, assimilate and reverberate multiple linguistic stimuli in more than one language across contexts ranging from academia to social media.

On the basis of the above, we performed an analysis of preliminary data derived from on an online questionnaire assessing the impact of health crisis on university students. Social interaction and language development of young adults is of great interest in linguistics, especially in Greece where young natives are considered to be poorly skilled in speaking their own language (neologisms, poor vocabulary, misspelling, syntactic and vocabulary mistakes) [6]. This study focused only on university students who are usually more prone to embracing neologisms, foreign words and concepts during a crisis and decelerate the process of their mental and language development. Furthermore, they demonstrate a greater potential to benefit from educational approaches and interventions. This study included answers by voluntary participants who were students in several universities in Greece. The data were collected anonymously from December 15th 2021 to January 10th 2022, during the outbreak of Omicron variant. The protocol was approved by the ethics committee of Aristotle University of Thessaloniki in accordance with the Declaration of Helsinki and all relevant guidelines and regulations. All participants attested informed consent before answering the questionnaire.

Social media groups and email lists of researchers were used to recruit participants. For the present analysis of preliminary data, as well as the final analysis only complete responses were taken into account. Complete responses were defined as those who of participants who answered all questions and sub-questions and successfully completed the questionnaire. Τhis questionnaire consisted of 5 principal domains with several sub-questions. The five main questions-domains were the following: (1) Have you observed changes in your language during pandemic concerning introduction of foreign terms/words? (2) Can you assess how many different foreign languages and phrases do you use every day? (3) Do you think that potential changes in the vocabulary of students will have an impact on their future linguistic competence? (4) If you were an educator, the teaching of which school lesson would you enhance? (5) Do you think that the health crisis will have a negative permanent impact on national languages after the pandemic ends? The null hypothesis was that linguistic alterations and changes in language during pandemic are not believed to be significant and this is obvious in each answer-domain. The chi square test was used to compare the percentage of each answer within questions. The G*Power software was utilized to calculate the sample size required to derive statistical significance. It was estimated that to reject the H0 hypothesis with a probability for Type I error: 0.80, and statistical significance level: 0.05, approximately 130 participants (at least) would be required.

In the present analysis, we present preliminary data concerning the main domains-questions based on the answers of the first 150 students. Οf 150 participants, 124 (82.6%; significance level: p < 0.01) observed foreign terms to be introduced in their everyday language (1st Qustion), with 128 (85.3%; p < 0.01) of them reporting that they have used terms coming from at least 3 different languages (2nd Question). More than half of them (79/150; 52.6%) agreed that the current changes to their vocabulary may have an impact on their future linguistic competence (3rd Question), and about one third (52/150; 34.6%) of them expressed fears that national languages will be negatively impacted by the situation. Nevertheless, more than 60% (94/150; 62.6%) disagreed with the latter and opted for enhancing biology and health literacy rather than language and expression in primary and secondary education curricula (p < 0.01).

To our knowledge, this is the first analysis to highlight the importance of ‘‘language crisis’’ along with health crisis. Α survey conducted by S. Charney et al. stated that pandemic influences pediatric speech and language development, and thus parents and clinicians should recognize this phenomenon and proactive in facilitating an optimal communication environment for children [7]. However, they referred only to children and did not include an analysis of real-world data. Recently, the World Youth Organization hosted an event to stand for ‘‘linguistics rights’’ highlighting that public health officials should provide information in a range of languages and provide public health information for indigenous people all over the world [8]. They concluded that the COVID-19 pandemic induces language discrimination and that linguistics rights are violated in countries where minority languages are spoken. No other study has been performed on this topic and the issue of how language changes during crises and adapts to new socioeconomic conditions remains largely unexplored. In this context, the results of our analysis add to the existing knowledge and raise concerns in the linguistic community.

Declined language skills of citizens can strongly affect the future of societies in many fields: educational, political, and even financial. The use of foreign words has been dramatically increased against national languages. This was also highlighted by the present analysis based on preliminary data of an observational study conducted in Greece. Our results showed that among Greek university students the vast majority has observed intrusion of many foreign terms in their everyday language vocabulary and is worried about how this could influence linguistic competence on a national level. Despite the fact that larger studies are needed to confirm these results, our study could lead to the conclusion that in linguistics researching factors with an impact on language can help identify real-world social, cultural and political phenomena and needs. Hence, the coronavirus outbreak and the new words and phrases born out of it and adopted by large populations worth more research attention in the foreseeable future.

## Data Availability

Data sharing not applicable to this article as no datasets were generated or analysed during the current study.
